# Risk Factors for Ebola Exposure in Health Care Workers in Boende, Tshuapa Province, Democratic Republic of the Congo

**DOI:** 10.1093/infdis/jiaa747

**Published:** 2020-12-03

**Authors:** Reena H Doshi, Nicole A Hoff, Anna Bratcher, Patrick Mukadi, Adva Gadoth, Bradly P Nicholson, Russell Williams, Daniel Mukadi, Matthias Mossoko, Joseph Wasiswa, Alexis Mwanza, Cyrus Sinai, Vivian H Alfonso, Rupal Shah, Matthew S Bramble, Benoit Ilunga-Kebela, Emile Okitolonda-Wemakoy, Jean Jacques Muyembe-Tamfum, Anne W Rimoin

**Affiliations:** Department of Epidemiology, University of California Los Angeles, Fielding School of Public Health, Los Angeles, California, USA; Department of Epidemiology, University of California Los Angeles, Fielding School of Public Health, Los Angeles, California, USA; Department of Epidemiology, University of California Los Angeles, Fielding School of Public Health, Los Angeles, California, USA; Institut National de Recherche Biomédicale, Kinshasa, Democratic Republic of the Congo; Faculté de Médecine, Université de Kinshasa, Kinshasa, Democratic Republic of the Congo; Department of Epidemiology, University of California Los Angeles, Fielding School of Public Health, Los Angeles, California, USA; Institue for Medical Research, Veterans Affairs Medical Center, Durham, North Carolina, USA; University of California Los Angeles-Democratic Republic of the Congo Research Program, Kinshasa, Democratic Republic of the Congo; Institut National de Recherche Biomédicale, Kinshasa, Democratic Republic of the Congo; Faculté de Médecine, Université de Kinshasa, Kinshasa, Democratic Republic of the Congo; Direction de Lutte Contre la Maladie, Ministère de la Santé Publique, Kinshasa, Democratic Republic of the Congo; University of California Los Angeles-Democratic Republic of the Congo Research Program, Kinshasa, Democratic Republic of the Congo; Direction de Lutte Contre la Maladie, Ministère de la Santé Publique, Kinshasa, Democratic Republic of the Congo; University of California Los Angeles-Democratic Republic of the Congo Research Program, Kinshasa, Democratic Republic of the Congo; Department of Epidemiology, University of California Los Angeles, Fielding School of Public Health, Los Angeles, California, USA; Department of Epidemiology, University of California Los Angeles, Fielding School of Public Health, Los Angeles, California, USA; Department of Epidemiology, University of California Los Angeles, Fielding School of Public Health, Los Angeles, California, USA; Department of Genetic Medicine Research, Children’s Research Institute, Children’s National Medical Center, Washington, District of Columbia, USA; Direction de Lutte Contre la Maladie, Ministère de la Santé Publique, Kinshasa, Democratic Republic of the Congo; Ecole de Sante Publique, Université de Kinshasa, Kinshasa, Democratic Republic of the Congo; Institut National de Recherche Biomédicale, Kinshasa, Democratic Republic of the Congo; Faculté de Médecine, Université de Kinshasa, Kinshasa, Democratic Republic of the Congo; Department of Epidemiology, University of California Los Angeles, Fielding School of Public Health, Los Angeles, California, USA

**Keywords:** Ebola, health care workers, risk factors, Democratic Republic of the Congo

## Abstract

**Background:**

Health care workers (HCW) are more likely to be exposed to Ebola virus (EBOV) during an outbreak compared to people in the general population due to close physical contact with patients and potential exposure to infectious fluids. However, not all will fall ill. Despite evidence of subclinical and paucisymptomatic Ebola virus disease (EVD), prevalence and associated risk factors remain unknown.

**Methods:**

We conducted a serosurvey among HCW in Boende, Tshuapa Province, Democratic Republic of Congo. Human anti-EBOV glycoprotein IgG titers were measured using a commercially available ELISA kit. We assessed associations between anti-EBOV IgG seroreactivity, defined as ≥2.5 units/mL, and risk factors using univariable and multivariable logistic regression. Sensitivity analyses explored a more conservative cutoff, >5 units/mL.

**Results:**

Overall, 22.5% of HCWs were seroreactive for EBOV. In multivariable analyses, using any form of personal protective equipment when interacting with a confirmed, probable, or suspect EVD case was negatively associated with seroreactivity (adjusted odds ratio, 0.23; 95% confidence interval, .07–.73).

**Discussion:**

Our results suggest high exposure to EBOV among HCWs and provide additional evidence for asymptomatic or minimally symptomatic EVD. Further studies should be conducted to determine the probability of onward transmission and if seroreactivity is associated with immunity.

Ebola virus disease (EVD) is a severe, often lethal disease that has led to substantial morbidity and mortality in sub-Saharan Africa [1]. Case fatality rates range from 50% to 90%, depending on the species and have historically been highest for Ebola virus (EBOV) [1, 2]. EBOV is considered to be a classic zoonosis and bats are the suspected reservoir, though this is unconfirmed [1]. Since 2000, the number of outbreaks and cases of EVD have increased substantially across the continent, due in part to rapid human population growth and increased contact with wildlife host species in previously untouched forest environments [1, 3].

Since the first reported outbreaks of EVD in humans in 1976, nosocomial infections have been an important driver of transmission, particularly among health care workers (HCWs) [4–7]. Nosocomial infections can easily occur in the absence of stringent protective measures, as human-to-human EBOV transmission generally occurs through contact with body fluids of symptomatic or deceased individuals [8]. HCWs are an estimated 21 to 32 times more likely to be infected with EBOV during an outbreak compared to people in the general adult population, due to close physical contact with patients and potential exposure to infectious fluids [9]. A survey conducted in and around Kikwit, in the Democratic Republic of the Congo (DRC), during the 1995 EBOV outbreak, found a 9% attack rate among HCWs [10]. During the 2014–2015 EVD outbreak in West Africa, at least 3% of EVD cases were among HCWs and of those, two-thirds died [9, 11]. The outbreak in North Kivu and Ituri in 2018–2020 resulted in 171 (5%) HCW infections, of which 44% died [12].

The difficulties associated with clinical recognition, lack of diagnostic capabilities, inadequate supply of personal protective equipment (PPE) such as gloves, gowns, and face shields, inadequate training, and poor public health infrastructure has led to challenges in the implementation of universal precautions to prevent exposure in resource-limited settings [9, 13]. Moreover, the consequences of EVD among HCWs can be significant and can lead to increased viral spread, particularly in the early stages. Additionally, significant losses in the workforce can lead to closure of health facilities and loss of routine services [4].

Although HCWs have represented a significant portion of EVD cases in past outbreaks, not all HCWs fall ill, despite frequent exposure to infectious patients. There is evidence for asymptomatic or paucisymptomatic EVD, that is few or mild symptoms, although the extent to which this occurs is unknown and estimates vary widely, ranging from 1.0% to 45.8% depending on the population, sampling method, location, time, and assay [14, 15]. A high prevalence of asymptomatic infection could have significant epidemiologic consequences, particularly if subclinical infections confer protective immunity [16].

The DRC has experienced 11 documented EVD outbreaks since its discovery in 1976 and is currently experiencing an outbreak in the province of Equateur, which was announced on 1 June 2020. In July 2014, during the massive West African EVD outbreak, DRC’s seventh EVD outbreak was confirmed near Boende, DRC. The etiologic agent was EBOV [17]. Between 26 July and 7 October 2014, a total of 68 EVD cases were reported (suspected, probably, and confirmed) and 38 were confirmed [18, 19]. Among the 68 cases, 11.6% (8) occurred in HCWs [17].

Following this outbreak, we conducted a serosurvey in November 2015 among HCWs providing care in Boende to improve our understanding of EBOV transmission dynamics. This study expands on previous work in a subset of this population to explore the seroprevalence EBOV using multiple assays [20]. We further investigated the occurrence of asymptomatic or paucisymptomatic forms of EVD and associated risk factors among HCW.

## METHODS

### Study Location

**Figure 1.**
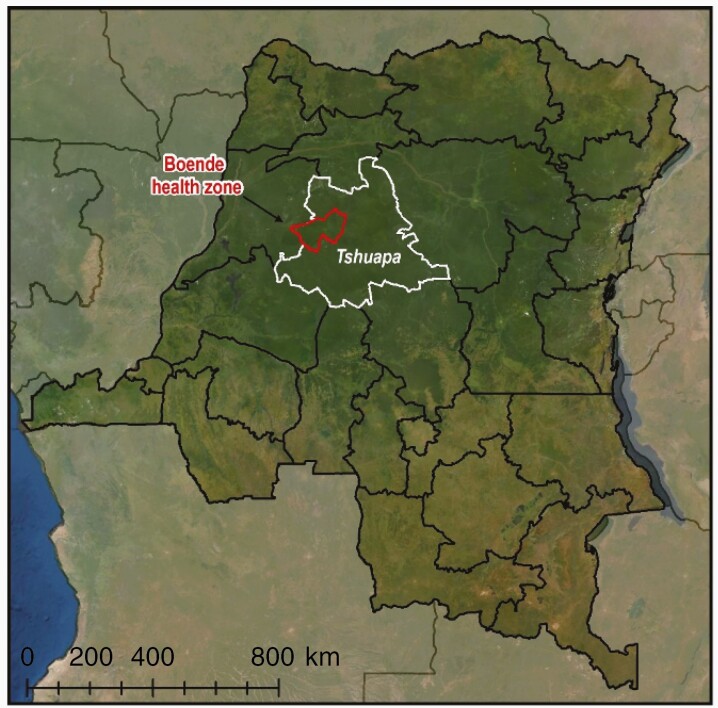
Location of the serosurvey in Boende town, Tshuapa province, Democratic Republic of the Congo.

Detailed methods are described elsewhere [20]. The serosurvey was conducted in Boende town, Tshuapa province (formerly Équateur province), in the northwestern part of the DRC (Figure 1). Boende has an estimated population of 45 000 and lies 70 km from Inkanamongo village, the location of the suspected index case, and 700 km northeast of Kinshasa, the capital city. Boende is surrounded by tropical rainforests and 2 large rivers.

### Study Population

Individuals were invited to participate if they were 18 years or older and were providing care to the local population during the time of the study. Additionally, all participants were healthy, that is presenting with no fever or illness at the time of enrollment, and not previously diagnosed with EVD. HCWs included those involved in both clinical and nonclinical care at health facilities, as well as informal care givers such as traditional healers and pastors who may not work in a standard health facility location.

### Study Procedures

All consenting individuals were interviewed and asked to provide a blood specimen by venipuncture in red-top vacutainer tubes (BD Biosciences) and undergo a basic physical assessment to obtain height, weight, and blood pressure measurements. A structured questionnaire was administered by trained interviewers in the participant’s preferred local language (French or Lingala). Data were collected using Open Data Kit Collect on a standard android tablet [21]. Interviewers documented demographics and potential exposure to EBOV virus in the community, health care facility, and via animals. Participants were compensated for transportation costs to and from the study site. After processing blood samples, aliquots of serum were frozen and shipped to the Institut National de Recherche Biomedicale for serological testing. Results were not provided to participants. Ethics approval was obtained from University of California Los Angeles Fielding School of Public Health and the Kinshasa School of Public Health.

### Serological Testing

Human anti-EBOV glycoprotein immunoglobulin G (GP IgG) titers were measured using a commercially available ELISA kit (Alpha Diagnostic International) following the manufacturer’s protocol. The methodology has been described elsewhere [20, 22]. Participants were considered seroreactive for this analysis if titers were greater than 2.5 units/mL. we chose a higher cutoff than the manufacturers cutoff of 1.0 units/ mL as other studies have suggested the utility of a more conservative cutoff [23, 24].

### Statistical Analyses

Basic descriptive statistics were calculated as frequencies and continuous variables were expressed as median and interquartile range (IQR). We assessed associations between anti-EBOV IgG seroreactivity, defined as ≥2.5 units/mL and risk factors using univariable logistic regression. Multivariable logistic regression analyses were used to identify community and occupational predictors for seroreactivity and were adjusted for age and sex. A 95% confidence interval (CI) that did not cross the null was considered to be evidence of an association. Variables of interest included receiving a blood transfusion, attending a funeral, having contact with human remains, participating in funeral rites, touching dead animals, traveling outside the province, frequenting markets, receiving an injection, visiting a health facility, receiving medication, and participating in active research or case finding activities. Occupational exposure data were gathered among HCWs who had interacted with confirmed, suspected, or probable Ebola cases. HCWs were classified based on their potential exposure to patients by reported occupation. Classifications were based on the World Health Organization (WHO) system of classification and have been described elsewhere [9, 20, 25]. Direct contact was defined as those with close contact to sick patients (eg, doctors, nurses, and traditional healers). Indirect contact was defined as contact with biological specimens, patient materials, or family members of sick patients (eg, laboratory technicians and room cleaners). Unlikely contact was defined as any position not directly related to clinical patient care (eg, hospital guards, administrators). We also considered use of PPE and hand washing as predictors for seroreactivity. Sensitivity analyses were explored using a more conservative definition of seroreactivity defined as ≥5 units/mL. All statistical analyses were carried out using SAS software, version 9.4 (SAS Institute).

## RESULTS

Among the 611 HCWs interviewed, 4 were EVD survivors and 25 had missing serologic data and were therefore excluded from the analysis. Demographics characteristics are presented in Table 1 for the remaining 582 HCWs. Overall, 22.5% were seroreactive for EBOV when considering the 2.5 units/mL cutoff. The median age among participants was 40 years (IQR, 31–50) and 49.5% were between the age of 18 and 39 years. The majority (64.4%) of participants were male, married or cohabitating (82.0%), and had finished primary school or secondary school (75.1%). Most participants reported being present for an EVD outbreak (91.1%); only 15.5% reported contact with a confirmed, probable, or suspected EVD case. Participants frequently had direct contact with patients in their current position (51.4%) and 28 (5.3%) believed they may have had EVD during the last outbreak but were never tested.

**Table 1.** Sample Characteristics of 582 Health Care Workers from Boende Health Zone in the Democratic Republic of the Congo, November 2015

Characteristic	No. (%)
Age, y, median (interquartile range)^a^	40 (31–50)
Unknown	4 (0.7)
18–39 y	288 (49.5)
40–59 y	247 (42.4)
60 y or older	43 (7.4)
Sex	
Male	375 (64.4)
Female	207 (35.6)
Education	
None	18 (3.1)
Started primary school	37 (6.4)
Finished primary school	212 (36.4)
Finished secondary school	225 (38.7)
Apprentice	4 (0.7)
College/university or graduate school	86 (14.8)
Civil status	
Single	84 (14.4)
Married or cohabitating	477 (82.0)
Divorced, separated, or widowed	21 (3.6)
Was ever present for an Ebola outbreak	
Yes	530 (91.1)
No	52 (8.9)
Has ever worked as a health care worker in an Ebola outbreak^b^	
Yes	432 (83.6)
No	85 (16.4)
Has ever had contact with a confirmed, probable, or suspected EVD case?	
Yes	90 (15.5)
No	471 (80.9)
Don’t know	21 (3.6)
Current health care worker type	
Nurse	171 (29.4)
Administrator	32 (5.5)
Room attendant	97 (16.7)
Hygiene service	84 (14.4)
Traditional healer or pastor	54 (9.3)
Red Cross worker	18 (3.1)
Midwife	43 (7.4)
Other^c^	83 (14.3)
Contact with patients in current position	
Direct	299 (51.4)
Indirect	201 (34.5)
No contact	82 (14.1)
Suspected they were infected with ebolavirus during the last outbreak^d^	
Yes	28 (5.3)
No	432 (81.5)
Don’t know	70 (13.2)
ADI GP result, units/mL	
0–1	266 (45.7)
> 1–2.5	185 (31.8)
> 2.5–5	96 (16.5)
> 5	35 (6.0)

Abbreviations: ADI GP, Alpha Diagnostic International glycoprotein; EVD, Ebola virus disease.

^a^Four participants did not know their age.

^b^Thirteen missing responses.

^c^The other category comprised physicians, epidemiologists, communication specialists, technicians, students, and maintenance workers.

^d^Fifty-two participants had not been present for an Ebola outbreak and were not eligible for the question.

In univariable analyses, being female (odds ratio [OR], 0.62; 95% CI, .40–.95) and university educated was negatively associated with seroreactivity (OR, 0.30; 95% CI, .13–.68) (Table 2). When stratifying by current position, administrators had 2.42 times the odds of being seroreactive (95% CI, 1.03–5.69) and traditional healers/pastors had 3.14 times the odds of being seroreactive (95% CI, 1.58–6.25) compared to nurses.

**Table 2.** Sample Characteristics by Seroreactivity (GP > 2.5) in 609 Health Care Workers from Boende Health Zone in the Democratic Republic of the Congo, November 2015

Characteristic	GP ≤ 2.5 units/mL, No. (%) n = 451	GP > 2.5 units/mL, No. (%) n = 131	Unadjusted Odds Ratio	95% Confidence Interval
Sex				
Male	280 (74.7)	95 (25.3)	Reference	
Female	171 (82.6)	36 (17.4)	0.62	.40–.95
Age, y, median (interquartile range)^a^	39 (31–50)	40 (30–49)	1.00	.98–1.01
18–39 y	224 (77.8)	64 (22.2)	Reference	
40–59 y	184 (74.5)	63 (25.5)	1.20	.80–1.79
60 y or older	39 (90.7)	4 (9.3)	0.36	.12–1.04
Education				
None	14 (77.8)	4 (22.2)	0.95	.30–3.01
Started primary school	25 (67.6)	12 (32.4)	1.60	.75–3.40
Finished primary school	156 (73.6)	56 (26.4)	1.19	.77–1.85
Finished secondary school	173 (76.9)	52 (23.1)	Reference	
Apprentice	4 (100.0)	0 (0.0)	…	
College/university or graduate school	79 (91.9)	7 (8.1)	0.30	.13–.68
Civil status				
Single	69 (82.1)	15 (17.9)	0.70	.39–1.27
Married or cohabitating	365 (76.5)	112 (23.5)	Reference	
Divorced, separated, or widowed	17 (81.0)	4 (19.0)	0.77	.25–2.33
Was ever present for an Ebola outbreak				
Yes	407 (76.8)	123 (23.2)	1.66	.76–3.63
No	44 (84.6)	8 (15.4)	Reference	
Has ever worked as a health care worker in an Ebola outbreak^b^				
Yes	339 (78.5)	93 (21.5)	1.28	.70–2.34
No	70 (82.4)	15 (17.6)	Reference	
Has ever had contact with a confirmed, probable, or suspected EVD case?				
Yes	74 (82.2)	16 (17.8)	0.69	.39–1.24
No	359 (76.2)	112 (23.8)	Reference	
Don’t know	18 (85.7)	3 (14.3)	0.53	.16–1.85
Current health care worker type				
Nurse	144 (84.2)	27 (15.8)	Reference	
Administrator	22 (68.8)	10 (31.3)	2.42	1.03–5.69
Room attendant	77 (79.4)	20 (20.6)	1.39	.73–2.63
Hygiene service	63 (75.0)	21 (25.0)	1.78	.94–3.38
Traditional healer or pastor	34 (63.0)	20 (37.0)	3.14	1.58–6.25
Red Cross worker	13 (72.2)	5 (27.8)	2.05	.68–6.23
Midwife	32 (74.4)	11 (25.6)	1.83	.83–4.08
Other	66 (79.5)	17 (20.5)	1.37	.70–2.69
Contact with patients in current position				
Direct	233 (77.9)	66 (22.1)	Reference	
Indirect	157 (78.1)	44 (21.9)	0.99	.64–1.52
No contact	61 (74.4)	21 (25.6)	1.22	.69–2.14
Suspected they were infected with Ebolavirus during the last outbreak^c^				
Yes	21 (75.0)	7 (25.0)	1.11	.46–2.68
No	332 (76.9)	100 (23.1)	Reference	
Don’t know	54 (77.1)	16 (22.9)	0.98	.54–1.79

Abbreviations: EVD, Ebola virus disease; GP, glycoprotein.

^a^Four participants did not know their age.

^b^Thirteen missing responses.

^c^Fifty-two participants had not been present for an Ebola outbreak and were not eligible for the question.

All community and occupational exposures were age- and sex-adjusted. Using any form of PPE when interacting with a confirmed, suspected, or probable EVD patient was negatively associated with seroreactivity (adjusted odds ratio [aOR], 0.23; 95% CI, .07–.73) (Table 3). When stratifying by type, only a face mask (aOR, 0.29; 95% CI, .09–.94) and gloves (aOR, 0.23; 95% CI, .07–.73) were associated with a decrease in odds of seroreactivity.

**Table 3.** Adjusted Odds Ratios of Seroreactivity (GP > 2.5) to Ebola GP by Possible Community Exposures to Ebolavirus Among 530 Health Care Workers in Boende Health Zone in the Democratic Republic of the Congo Who Have Been Present at an Ebola Outbreak, November 2015

Exposure	Adjusted Odds Ratio^a^	95% Confidence Interval
Activities performed during their last Ebola outbreak		
Received a blood transfusion	…	.07–5.14
Attended a funeral	1.11	.65–1.89
Had direct exposure to human remains	0.72	.27–1.97
Participated in funeral rites	1.24	.69–2.26
Came in contact with dead animals	1.33	.53–3.30
Traveled outside your locality	0.85	.52–1.40
Frequented markets	0.70	.46–1.05
Received an injection	1.01	.39–2.58
Went to a health facility for an ailment	0.50	.21–1.22
Took medication	0.99	.58–1.69
Active research (searching for cases in community)	…	
Activities performed on a confirmed, suspected, or probable Ebola patient^b^		
Been in the patient’s room	0.79	.22–2.83
Performed examinations (clinical or laboratory)	0.86	.17–4.44
Given food to a patient	1.13	.32–3.99
Conversed with a patient	3.80	.73–19.83
Washed the patient’s clothes	0.99	.10–10.41
Had contact with patient’s bodily fluids	2.39	.79–7.30
Washed a cadaver	1.28	.13–12.76
Cleaned patient’s room	1.40	.34–5.83
Participated in funeral rites/rituals	1.94	.52–7.19
Used any PPE when interacting with a confirmed, suspected, or probable Ebola patient^b^	0.23	.07–.73
Type of PPE used^b^		
Face mask	0.29	.09–.94
Laboratory coat	0.50	.15–1.62
Gown	0.45	.15–1.39
Gloves	0.23	.07–.73
Respirator	0.31	.06–1.50
Washed hands after each contact with a confirmed, suspected, or probable Ebola patient^b^	1.75	.94–3.28

^a^Adjusted for age and sex.

^b^n = 90, the number of HCW who had contact with a confirmed, suspected, or probable EVD case.

In the sensitivity analyses, similar trends were observed when a more conservative cutoff for seroreactivity (≥5 units/mL) was used. Results are presented in Supplementary Table 1 and Supplementary Table 2.

## Discussion

Our results suggest high exposure to EBOV among HCWs in Boende without a history of diagnosed EVD. Using a ≥2.5 units/mL cutoff, 22.5% of surveyed participants were seroreactive, which is consistent with other studies [23, 26, 27]. A 2016 meta-analysis estimated proportions of asymptomatic Ebola infections at 27.1% (95% CI, 14.5%–39.6%) [28]. Individual serostudies in areas surrounding EVD outbreaks commonly find seroprevalence of Ebola antibodies in individuals who have no history of EVD diagnosis. In 1976, in Sudan, the WHO found that 19% of contacts of individuals with EVD had antibodies to the virus [29]. Estimates from other studies are lower; for example, a serologic survey conducted during the 1995 EVD outbreak in Kikwit found an average seroprevalence of 9.3% in surrounding villages [30]. In addition to areas surrounding EVD outbreaks, this phenomenon has also been observed in areas with no record of EVD cases. A serostudy in the Sankuru province in DRC found that 11% of the study population was seropositive for EBOV despite no known outbreaks having occurred in the area [31]. Finally, a study in the northeastern region of the DRC reported an EBOV seroprevalence of 18.7% in the indigenous Efe (pygmy) population [27]. Collectively, despite varying study methods and serological tests, the results imply high seroprevalence of EBOV antibodies among individuals with no recollection of an EVD-like illness in various parts of the DRC and sub-Saharan Africa.

We would expect to see higher rates of exposure in an HCW population such as the one surveyed here when compared to the general population. HCWs are considered at higher risk of EVD due to their close contact with patients and many in our sample reported involvement in the 2014 EVD response [20, 32]. While it is likely that some of the participants were exposed to EBOV while working during the outbreak, we cannot confirm when and where exposure may have occurred. It is possible that exposure could have happened outside of the workplace during the 2014 outbreak or be unrelated to the 2014 EVD outbreak. Other studies suggest that filoviruses may circulate in the environment without any severe clinical manifestations [33–35]. Boende and the surrounding areas are rural and the population mostly subsists on hunting and gathering. These characteristics make Boende at risk for zoonotic spillover of EBOV, which may have contributed to our observed seroreactivity outside of the official 2014 outbreak. Additionally, we cannot rule out the possibility of the presence of an unidentified EBOV strain with low pathogenicity and cross-reactivity with our antibody assay. However, serologic response to other EBOV proteins, including nucleoprotein, viral protein 40, and EBOV GP-bearing human immunodeficiency virus pseudotype viruses was documented in a subset of this population. Approximately 11.7% of the population was considered reactive to more than one test, and 2.8% demonstrated pseudoneutralization ability [20]. Outside of the infection, it is possible that seroreactivity was generated through low viral inocula, inactivated virus, isolated viral antigens in the workplace, or while gathering or consuming bushmeat or other foods [36].

It is impossible to determine if these HCWs were asymptomatic or paucisymtomatic. The initial symptoms associated with EVD are nonspecific and mirror common diseases in DRC, such as typhoid and malaria, thus may not initially be recognizable as EVD [37]. It is possible that EBOV could be transmissible to others from individuals with mild symptoms, although this may be rare [38, 39]. Our findings do not indicate if exposure is associated with immunity. This analysis, along with further research on the association between seroreactivity and immunity, could identify HCWs who are naturally immune to EVD. During future outbreaks, HCWs could act as caregivers in Ebola treatment centers and may reduce the stigma associated with utilizing survivors. Additionally, a safe and effective Ebola vaccine is now licensed and will likely be considered for preventative use in high-risk populations such as HCWs [40, 41]. As with other vaccine-preventable diseases, understanding preexisting immunity and the proportion of the population at risk will be critical to determine what vaccine coverage rates are needed for effective outbreak prevention and control.

While the HCWs in our study, as a whole, showed high exposure to EBOV, specific subgroups were associated with higher odds of EBOV seroreactivity. Being a traditional healer or pastor was associated with increased odds of seroreactivity compared to nurses. Traditional medicine is common throughout sub-Saharan African and in the DRC, with the number of traditional healers surpassing doctors and nurses in many rural areas [42–44]. Thus, in many settings, traditional healers are likely to be the first source of treatment when someone falls ill, but often do not have PPE or other medical resources to treat EVD patients safely, leading to exposure [45]. Additionally, those in administrative roles are also associated with increased odds of seroreactivity compared to nurses. We hypothesize that these individuals might come in contact with EVD patients unknowingly, as patients at health facilities seeking care, without wearing proper PPE or following consistent hand washing practices. Alternatively, this increase could be explained by a community-level exposure independent of their profession. Not surprisingly, those who used any PPE showed significantly reduced odds of seroreactivity in the multivariable model. Furthermore, both use of a facemask and gloves were independently associated with lower odds of seroreactivity in the multivariable model.

Demographic risk factors for EBOV infection and EVD, such as age, sex, and ethnicity, are not well characterized [46]. While age was not a significant predictor of seroreactivity, being female was associated with lower odds compared to being male, a pattern that has been documented previously [31]. This might be explained by a male dominated workforce and therefore an increased risk of exposure to EBOV in this nontraditional population.

Our study is subject to a number of limitations. This is a cross-sectional study targeting a specific population and therefore the results may not be generalizable to the general population. We attempted to enroll all HCWs in Boende health zone; however, without a comprehensive list of HCWs, it was impossible to determine exact participation rates in our study. Additionally, we interviewed study participants a year after the outbreak, and so there is a possibility of misclassification due to recall inaccuracies, particularly for questions on exposure during the outbreak. However, we would expect misclassification to be nondifferential and unrelated to serologic results. We note that a small number of participants suspected that they had become infected with EVD, although none were tested, and only 7 of these 28 were seroreactive. Exclusion of these individuals in sensitivity analyses had no effect on our results.

To date, there is no gold standard serologic test for EBOV and no assay has been approved by the Food and Drug Administration. Previous work has shown that seroreactivity is difficult to define and serologic tests may often overestimate seroreactivity, thus we must be cautious of the interpretations [15, 20]. We used a commercially available test and considered various cutoffs for seroreactivity. While we choose to use a higher cutoff than the manufacturer’s suggestions (1.0 units/mL), other studies suggest a more conservative cutoff may be warranted due to the potential for cross-reactivity and high background [23, 24]. We conducted sensitivity analyses with a higher cutoff to account for these concerns and overall seroprevalence in this population was reduced to 6%. Similar associations were observed and are presented in Supplementary Table 1 and Supplementary Table 2.

Our results provide additional evidence for asymptomatic or paucisymptomatic EVD in the DRC and sub-Saharan Africa. Further studies to distinguish between asymptomatic or paucisymptomatic infections should be conducted along with studies to determine whether these individuals are infectious and if seroreactivity is associated with immunity. Additionally, more detailed analyses looking at additional risk factors should be considered. Regardless, high EBOV seroreactivity in HCWs underscores the importance of appropriate infection prevention control practices and education in health care settings to prevent nosocomial disease transmission of Ebola and other communicable diseases.

## Supplementary Data

Supplementary materials are available at *The Journal of Infectious Diseases* online. Consisting of data provided by the authors to benefit the reader, the posted materials are not copyedited and are the sole responsibility of the authors, so questions or comments should be addressed to the corresponding author.

jiaa747_Suppl_Supplementary_Table_1

jiaa747_Suppl_Supplementary_Table_2

## Notes

***Acknowledgments.*** Many individuals contributed to the design of this study and data collection in the field. We thank the health care works of the Tshuapa province for their participation. We also thank, Dr Ado Bwaka, Jose Ngamboli, Nathalie Kavira, Rachel Mutombe, and the DRC Ministry of Health for their assistance with field logistics and the implementation of this study; and Dr Tito Serafini and Dr Guy Cavet of Atreca, Inc, for their support of this research.

***Financial support.*** This work was supported by the Bill and Melinda Gates Foundation grants OPP1066534 and OPP1195609 and the Faucett Catalyst Fund.

***Potential conflicts of interest.*** All authors: No reported conflicts of interest. All authors have submitted the ICMJE Form for Disclosure of Potential Conflicts of Interest. Conflicts that the editors consider relevant to the content of the manuscript have been disclosed.

Presented in part: Eighth International Symposium on Filoviruses, Antwerp, Belgium, 13 September 2016; and 65th Annual Meeting of the American Society of Tropical Medicine and Hygiene, Atlanta, Georgia, 17 November 2016.
