# Supplementary material for: Risk Factors for Ebola Exposure in Health Care Workers in Boende, Tshuapa Province, Democratic Republic of the Congo
Source: J Infect Dis. 2020 Dec 3;226(4):608–15. doi: 10.1093/infdis/jiaa747 (PMC9441197; doi:10.1093/infdis/jiaa747)
Supplement: jiaa747_Suppl_Supplementary_Table_1 [file jiaa747_suppl_supplementary_table_1.docx]

**Supplemental Table 1.** Sample Characteristics by seroreactivity (GP > 5) in 582 healthcare workers from Boende health zone in the Democratic Republic of the Congo, November 2015

|  | GP < 5  n=547 | | GP > 5  n=35 | |  |  |
| --- | --- | --- | --- | --- | --- | --- |
|  | (n) | (%) | (n) | (%) | Unadjusted Odds Ratio | 95% Confidence Interval |
| Sex |  |  |  |  |  |  |
| Male | 346 | 92.3 | 29 | 7.7 | reference |  |
| Female | 201 | 97.1 | 6 | 2.9 | 0.36 | 0.15, 0.87 |
| Age^a^ [median (IQR)] | 39 (31,50) | | 41 (30-52) | | 1.01 | 0.98, 1.03 |
| 18-39 | 274 | 95.1 | 14 | 4.9 | reference |  |
| 40-59 | 227 | 91.9 | 20 | 8.1 | 1.72 | 0.85, 3.49 |
| 60 or older | 42 | 97.7 | 1 | 2.3 | 0.47 | 0.06, 3.64 |
| Education |  |  |  |  |  |  |
| None | 17 | 94.4 | 1 | 5.6 | 0.63 | 0.08, 5.06 |
| Started primary school | 35 | 94.6 | 2 | 5.4 | 0.62 | 0.14, 2.78 |
| Finished primary school | 200 | 94.3 | 12 | 5.7 | 0.65 | 0.31, 1.38 |
| Finished secondary school | 206 | 91.6 | 19 | 8.4 | reference |  |
| Apprentice | 4 | 100.0 | 0 |  | - |  |
| College/University or Graduate school | 85 | 98.8 | 1 | 1.2 | 0.13 | 0.02, 0.97 |
| Civil status |  |  |  |  |  |  |
| Single | 81 | 96.4 | 3 | 3.6 | 0.51 | 0.15, 1.71 |
| Married or cohabitating | 445 | 93.3 | 32 | 6.7 | reference |  |
| Divorced, separated, or widowed | 21 | 100.0 | 0 |  | - |  |
| Was ever present for an Ebola outbreak |  |  |  |  |  |  |
| Yes | 497 | 93.8 | 33 | 6.2 | reference |  |
| No | 50 | 96.2 | 2 | 3.8 | 1.66 | 0.39, 7.12 |
| Has ever worked as a healthcare worker in an Ebola outbreak^b^ |  |  |  |  |  |  |
| Yes | 404 | 93.5 | 28 | 6.5 | reference |  |
| No | 82 | 96.5 | 3 | 3.5 | 1.89 | 0.56, 6.38 |
| Has ever had contact with a confirmed, probable, or suspected EVD case? |  |  |  |  |  |  |
| Yes | 84 | 93.3 | 6 | 6.7 | 1.18 | 0.47, 2.93 |
| No | 444 | 94.3 | 27 | 5.7 | reference |  |
| Don't know | 19 | 90.5 | 2 | 9.5 | 1.73 | 0.38, 7.82 |
| Current healthcare worker type |  |  |  |  |  |  |
| Nurse | 165 | 96.5 | 6 | 3.5 | reference |  |
| Administrator | 28 | 87.5 | 4 | 12.5 | 3.93 | 1.04, 14.81 |
| Room Attendant | 91 | 93.8 | 6 | 6.2 | 1.81 | 0.57, 5.79 |
| Hygienic Service | 74 | 88.1 | 10 | 11.9 | 3.72 | 1.30, 10.61 |
| Traditional Healer or Pastor | 49 | 90.7 | 5 | 9.3 | 2.81 | 0.82, 9.59 |
| Red Cross Worker | 16 | 88.9 | 2 | 11.1 | 3.44 | 0.64, 18.46 |
| Midwife | 42 | 97.7 | 1 | 2.3 | 0.66 | 0.78, 5.59 |
| Other | 82 | 98.8 | 1 | 1.2 | 0.34 | 0.04, 2.83 |
| Contact with patients in current position |  |  |  |  |  |  |
| Direct | 285 | 95.3 | 14 | 4.7 | reference |  |
| Indirect | 185 | 92.0 | 16 | 8.0 | 1.76 | 0.84, 3.69 |
| No contact | 77 | 93.9 | 5 | 6.1 | 1.32 | 0.46, 3.78 |
| Suspected they were infected with Ebolavirus during the last outbreak^c^ |  |  |  |  |  |  |
| Yes | 24 | 85.7 | 4 | 14.3 | 2.83 | 0.91, 8.82 |
| No | 408 | 94.4 | 24 | 5.6 | reference |  |
| Don't know | 65 | 92.9 | 5 | 7.1 | 1.31 | 0.48, 3.55 |
