# Supplementary material for: Risk Factors for Ebola Exposure in Health Care Workers in Boende, Tshuapa Province, Democratic Republic of the Congo
Source: J Infect Dis. 2020 Dec 3;226(4):608–15. doi: 10.1093/infdis/jiaa747 (PMC9441197; doi:10.1093/infdis/jiaa747)
Supplement: jiaa747_Suppl_Supplementary_Table_2 [file jiaa747_suppl_supplementary_table_2.docx]

Supplemental Table 2: Adjusted odd ratios of seroreactivity (GP > 5) to Ebola GP by possible community exposures to Ebolavirus among 530 healthcare workers in Boende health zone in the Democratic Republic of the Congo who have been present at an Ebola outbreak, November

|  | Adjusted Odds Ratio* | 95% Confidence Interval |
| --- | --- | --- |
| Performed these activities during their last Ebola outbreak: |  |  |
| *Received a blood transfusion* | - |  |
| *Attended a funeral* | 2.10 | 0.95, 4.68 |
| *Had direct exposure to human remains* | 1.14 | 0.26, 5.08 |
| *Participated in funeral rites* | 1.91 | 0.80, 4.68 |
| *Came in contact with dead animals* | 2.26 | 0.63, 8.12 |
| *Traveled outside your locality* | 1.22 | 0.55, 2.73 |
| *Frequented markets* | 1.01 | 0.49, 2.04 |
| *Received an injection* | 0.67 | 0.09, 5.13 |
| *Went to a health facility for an ailment* | 0.34 | 0.05, 2.54 |
| *Took medication* | 1.34 | 0.56, 3.20 |
| *Active research (searching for cases in community)* | - |  |
| Activities performed on a confirmed, suspected, or probable Ebola patient**: |  |  |
| *Been in the patient's room* | 1.45 | 0.16, 13.44 |
| *Performed Examinations (clinical or laboratory)* | - |  |
| *Given food to a patient* | 0.54 | 0.06, 5.15 |
| *Conversed with a patient* | 1.99 | 0.20, 19.54 |
| *Washed the patients clothes* | 2.49 | 0.19, 31.90 |
| *Had contact with patient's bodily fluids* | 2.17 | 0.40, 11.73 |
| *Washed a cadaver* | 3.37 | 0.28, 40.71 |
| *Cleaned patient's room* | 2.92 | 0.46, 18.52 |
| *Participated in funeral rites/rituals* | 0.93 | 0.10, 8.84 |
| Used any PPE when interacting with a confirmed, suspected, or probable Ebola patient** | 0.39 | 0.07, 2.26 |
| Type of PPE used**: |  |  |
| *Face mask* | 0.30 | 0.05, 1.84 |
| *Lab coat* | 0.48 | 0.08, 2.96 |
| *Gown* | 0.81 | 0.15, 4.36 |
| *Gloves* | 0.39 | 0.07, 2.26 |
| *Respirator* | 0.41 | 0.04, 3.86 |
| Washed hands after each contact with a confirmed, suspected, or probable Ebola patient** | 1.73 | 0.70, 4.25 |
| *Adjusted for age and sex | | |
| **n=90, the number of HCW who had contact with a confirmed, suspected or probable EVD case | | |
